# Geometry of static $w=-1/5$ perfect fluid spheres in general relativity

**DOI:** 10.1140/epjc/s10052-022-10349-2

**Published:** 2022-04-25

**Authors:** Behnaz Fazlpour, Ali Banijamali, Valerio Faraoni

**Affiliations:** 1 Department of Physics, Babol Branch, Islamic Azad University, Babol, Iran; 2 Department of Basic Sciences, Babol Noshirvani University of Technology, Babol, Iran; 3 Department of Physics and Astronomy, Bishop’s University, 2600 College Street, Sherbrooke, QC J1M 1Z7 Canada

## Abstract

We discuss the physical features of two recent classes of analytical solutions of the Einstein equations sourced by an exotic perfect fluid with equation of state $P=-\rho /5$. These geometries depend on up to four parameters and are static and spherically symmetric. They describe compact spaces with naked central singularities.

## Introduction

Recently, two new families of static and spherically symmetric solutions of the Einstein equations (without cosmological constant) were proposed by Semiz [1]. The matter source is a perfect fluid with constant barotropic equation of state $P =-\rho /5$, where $\rho$ and *P* are the fluid energy density and pressure, respectively [1]. One would like to understand the physical nature of these solutions and assess whether they can be useful to model regions of stars, at least as toy models. The equation of state $P=-\rho /5$ is clearly unphysical, as one would be hard put to find realistic situations described by this fluid, but dark energy-like stars (and even phantom energy stars [2]) have been studied in the literature [3–10], as well as halos of exotic energy [11]. Although dark energy has pressure $P<-\rho /3$ and there are all indications that, if it is responsible for the present acceleration of our universe, it has equation of state $P \simeq -\rho$ [12], our situation with $P=-\rho /5$ could still serve as a toy model for hypothetical objects formed by a negative pressure fluid. Moreover, from the mathematical point of view, simple solutions of the Einstein equations describing perfect fluids are relatively difficult to find. Although there are over one hundred analytical solutions of the Einstein equations sourced by perfect (and even imperfect) fluids that constitute potential candidates to model relativistic stars, or at least stellar regions [13, 14], almost all of them turn out to be unphysical for one reason or another [14]. Here we examine the new solutions of [1] to understand their physical features (or lack thereof). These geometries are written in Buchdahl coordinates but it is more instructive from the physical point of view to rewrite them in terms of Schwarzschild-like coordinates, which we do here.

We follow the notation of Ref. [15]: the metric signature is ${-}{+}{+}{+}$ and we use units in which the speed of light in vacuo *c* and Newton’s constant *G* are unity, while $\kappa \equiv 8\pi G$ to keep with Ref. [1].

Semiz’s proposal consists of a four-parameter family of solutions of the Einstein equations with zero cosmological constant

1 $$\begin{aligned} {\mathcal {R}}_{ab}-\frac{1}{2} \, g_{ab} {\mathcal {R}} = \kappa \,T_{ab} \,, \end{aligned}$$

where ${\mathcal {R}}_{ab}$ is the Ricci tensor of the metric $g_{ab}$ and ${\mathcal {R}}$ is the Ricci scalar. The matter source is a perfect fluid with stress-energy tensor

2 $$\begin{aligned} T_{ab}= \left( P+\rho \right) u_a u_b +P g_{ab} \,, \end{aligned}$$

where $u^a$ is the fluid 4-velocity and the equation of state is $P=-\rho /5$ [1]. These geometries are spherically symmetric and static in the appropriate coordinate range. There are two new classes of solutions in [1]: the most general family is parametrized by four constants $\left( C_0, C_1, C_2, C_3 \right)$ and has line element

3 $$\begin{aligned} ds^2= & {} -\frac{3C_1 \left( C_0 +C_1r \right) }{f(r)} \, dt^2 +\frac{f(r)}{3C_1 \left( C_0+C_1r \right) } \, dr^2 \nonumber \\&\quad + \frac{f^2(r)}{9C_1^2} \, d\varOmega _{(2)}^2 \,, \end{aligned}$$

with $C_1\ne 0$, $C_0 +C_1r \ne 0$, and where

4 $$\begin{aligned} f(r) = 3\left( C_1 C_2 +r \right) +C_3 \left( C_0 +C_1r \right) ^3 \,, \end{aligned}$$

while $d\varOmega _{(2)}^2 \equiv d\vartheta ^2 + \sin ^2 \vartheta \, d\varphi ^2$ is the line element on the unit 2-sphere. The energy density is [1]

5 $$\begin{aligned} \rho (r) = -5P(r)= \frac{-45 \kappa \, C_1^3 C_3 \left( C_0 +C_1 r \right) ^2}{f^2(r)} \end{aligned}$$

and is non-negative provided that

6 $$\begin{aligned} C_1 C_3 \le 0 \,, \end{aligned}$$

which we assume in the following, while the limiting situation given by $C_3=0$ corresponds to vacuum. The solution for $C_1=0$ is not obtained continuously from Eqs. (3) and (5) in the limit $C_1\rightarrow 0$ but requires a separate discussion [1]. This second family is parametrized by the remaining three constants [1]: we begin by analyzing this second family (or “special solution” in the nomenclature of [1]) in the following section.

## Special solution $C_1=0$

This 3-parameter $\left( C_0, C_2, C_3 \right)$ family of solutions is described by the line element [1]

7 $$\begin{aligned} ds^2=-\frac{C_0^2}{g(r)} \, dt^2 + \frac{g(r)}{C_0^2} \, dr^2 + \frac{g^2(r)}{C_0^2} \, d\varOmega _{(2)}^2 \end{aligned}$$

with $C_0\ne 0$ and where

8 $$\begin{aligned} g(r)= & {} C_0\left( C_2+C_3r\right) -r^2 \end{aligned}$$

9 $$\begin{aligned} \rho (r)= & {} -5P(r) = \frac{5 \kappa \, C_0^2}{g^2(r)} \,. \end{aligned}$$

In order to preserve the metric signature it must be $g(r)>0$ (if *g*(*r*) becomes negative, the coordinates *t* and *r* switch their timelike and spacelike natures, as in the Schwarzschild geometry at the horizon $r=2m$).

We rewrite the line element (7) in terms of the areal radius $R(r) = g(r)/|C_0|$. This relation is inverted by first obtaining

10 $$\begin{aligned} r^2 -C_0 C_3 r + \left( |C_0| R-C_0 C_2\right) =0 \end{aligned}$$

and solving for

11 $$\begin{aligned} r (R) = \frac{1}{2} \left( C_0 C_3 \pm \sqrt{ C_0^2 C_3^2 +4 \left( C_0 C_2 -|C_0| R\right) } \right) \,. \end{aligned}$$

The argument of the square root in the right-hand side must be non-negative to keep *r* real, which gives the limitation

12 $$\begin{aligned} 0 \le R < \frac{ C_0^2 C_3^2+4 C_0 C_2}{4|C_0|} \equiv R_\mathrm {max} \end{aligned}$$

on the range of the areal radius. The latter begins from zero at $r_1=\frac{1}{2}\left( C_0 C_3-\sqrt{C_0^2 C_3^2+4C_0 C_2} \right)$, increases to the maximum

13 $$\begin{aligned} R_\mathrm {max} = R \left( \frac{C_0 C_3}{2} \right) \,, \end{aligned}$$

and then decreases until it vanishes again at $r_2=\frac{1}{2}\left( C_0 C_3+\right.$ $\left. \sqrt{C_0^2 C_3^2+4C_0 C_2}\right)$. The two coordinate charts $r_1\le r \le C_0 C_3/2$ and $C_0 C_3/2 \le r \le r_2$ cover the same physical region $0\le R \le R_\mathrm {max}$. We restrict ourselves to $r_1\le r \le C_0 C_3/2$, in which $dR/dr>0$, by choosing the negative sign in Eq. (2.5).

We write

14 $$\begin{aligned} C_0 C_3-2r = \mp \sqrt{ C_0^2 C_3^2 +4\left( C_0 C_2 -|C_0|R\right) } \end{aligned}$$

and, substituting the relation between differentials

15 $$\begin{aligned} dr= \frac{ |C_0|}{C_0C_3-2r} \, dR \end{aligned}$$

and using Eq. (14), the line element (7) becomes

16 $$\begin{aligned} ds^2 = -\frac{|C_0|}{R} \, dt^2 + \frac{dR^2}{ 4\left( \frac{R_\mathrm {max}}{R} -1\right) } +R^2 d\varOmega _{(2)}^2 \,. \end{aligned}$$

The equation $\nabla ^c R \nabla _c R =g^{RR}= 0$ locating the apparent horizons (see, e.g., [16]) has $R_\mathrm {max}$ as the only root, which is a single root and therefore there are no apparent horizons for $R<R_\mathrm {max}$ (we discuss the physical meaning of the formal root $R_\mathrm {max}$ below).

The energy density [1]

17 $$\begin{aligned} \rho (R) = \frac{5 \kappa \, C_0^2}{g^2(r)}= \frac{5\kappa }{R^2} \end{aligned}$$

and the pressure $P=-\rho /5$ (which are always non-zero) diverge at the origin $R = 0$, which corresponds to $r=r_1$, together with the Ricci scalar

18 $$\begin{aligned} {\mathcal {R}}= -\kappa \, T = \kappa \left( \rho -3P \right) =\frac{8\kappa }{5} \, \rho = \frac{8\kappa ^2}{R^2} \,, \end{aligned}$$

therefore there is a naked spacetime singularity at the origin $R=0$.

The Misner-Sharp-Hernandez mass $M_\mathrm {MSH}(R)$ defined in spherical symmetry by [17, 18]

19 $$\begin{aligned} 1-\frac{2M_\mathrm {MSH}}{R} = \nabla ^c R \nabla _c R = g^{RR} \end{aligned}$$

reads

20 $$\begin{aligned} M_\mathrm {MSH} (R) = \frac{1}{2} \left( 5R-4R_\mathrm {max}\right) \end{aligned}$$

for the geometry (16) and is negative in the region $0<R< 4R_\mathrm {max}/5$ around the naked singularity. This fact is not surprising: it has been argued that the Misner-Sharp-Hernandez mass (to which the Hawking-Hayward quasilocal mass [19, 20] reduces in spherical symmetry [21]) is misbehaved near naked singularities, Cauchy horizons, or regions with the wrong asymptotic flatness [22, 23]. This is the case, for example, for the inner region of the Reissner–Nordström black hole near the Cauchy horizon, for the entire Schwarschild spacetime with negative mass, and for the Fisher–Janis–Newman–Winicour–Buchdahl–Wyman scalar field solution of the Einstein equations [24–31] for the parameter values for which there is a naked singularity [32].

Let us come to the maximum value $R_\mathrm {max}$ of the areal radius which, in spite of being a formal root of the equation $\nabla ^cR \nabla _c R=0$, does not describe a horizon but is instead the antipode of the origin $R=0$ in a compact space. To see this fact, it is instructive to study the behaviour of radial null geodesics in this geometry. Consider the outgoing $(+)$ and ingoing $(-)$ congruences of radial null geodesics with tangents $l_{(\pm )}^{\mu } =dx^{\mu }/d\lambda$, where $\lambda$ is an affine parameter along these curves. These tangents have components $l_{(\pm )}^\mu = \left( l^0, l^1, 0, 0\right)$ and the normalization $l^{(\pm )}_{a} l_{(\pm )}^a=0$ yields

21 $$\begin{aligned} l_{(\pm )}^1 = \pm \frac{2}{R}\sqrt{\left( R_\mathrm {max}-R\right) |C_0|} \, l_{(\pm )}^0 \,; \end{aligned}$$

since a null vector can be rescaled by a function, we can choose $l^0 =1$ (which means choosing the coordinate time *t* as the affine parameter along these null geodesics), obtaining

22 $$\begin{aligned} l_{(\pm )}^\mu = \left( 1, \pm \frac{2}{R}\sqrt{\left( R_\mathrm {max}-R\right) |C_0|}, 0, 0 \right) \,. \end{aligned}$$

We then have the first order equations

23 $$\begin{aligned}&\frac{dt}{d\lambda }=1 \end{aligned}$$

24 $$\begin{aligned}&\frac{R}{ \sqrt{R_\mathrm {max}-R}} \,\frac{d R}{d\lambda }=\pm 2\sqrt{|C_0|}\,, \end{aligned}$$

which integrate to

25 $$\begin{aligned}&t(\lambda ) = \lambda -\lambda _{0}\,, \end{aligned}$$

26 $$\begin{aligned}&\sqrt{R_\mathrm {max}-R} \left( R +2R_\mathrm {max}\right) = \mp 3\sqrt{|C_0|}\left( \lambda -\lambda _{0}\right) \end{aligned}$$

where $\lambda _0$ is an integration constant. Unfortunately this relation cannot be inverted explicitly.

**Fig. 1:**
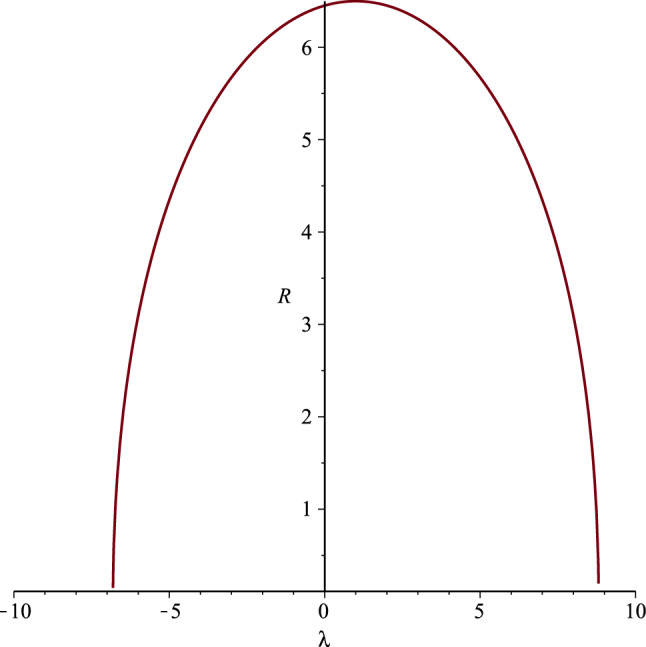
The areal radius *R* versus the affine parameter $\lambda$ along the radial null geodesics of the geometry (16), for the parameter values $C_{0}= C_{2}=2$, $C_{3}=3$, and $\lambda _{0}=1$. Outgoing geodesics slow down as they approach $R_\mathrm {max}$, where they stop. Ingoing geodesics starting near $R_\mathrm {max}$ do so extremely slowly but accelerate as they approach $R=0$

Since

27 $$\begin{aligned} \frac{dR}{dt} =\frac{dR}{d\lambda } = \pm 2\sqrt{|C_0|} \, \frac{ \sqrt{ R_\mathrm {max}-R}}{R} \end{aligned}$$

(with the upper sign for outgoing and the lower one for ingoing radial geodesics), near the origin $R=0$ it is $dR/dt \sim +\infty$ for outgoing and $dR/dt \sim -\infty$ for ingoing geodesics. Furthermore, $dR/dt=0$ at $R=R_\mathrm {max}$. Outgoing radial null geodesics starting near the origin do so extremely fast but they slow down as they approach the maximum possible radius $R_\mathrm {max}$, which can only be reached with zero velocity (see Fig. 1). A null geodesic starting exactly at $R_\mathrm {max}$ does so with zero velocity $dR/d\lambda$ and remains there. Ingoing radial null geodesics starting near the maximum radius $R_\mathrm {max}$ are slow and accelerate as they get closer to the central naked singularity, which they approach with infinite velocity $dR/d\lambda \rightarrow -\infty$.

We can also study radial timelike geodesics with tangents $u^{\mu } =\left( u^0, u^1, 0, 0 \right)$. The normalization $u^c u_c=-1$ gives

28 $$\begin{aligned} u^1 = \pm \frac{2}{\sqrt{R}} \sqrt{ \left( R_\mathrm {max}-R \right) \left[ \frac{ |C_0| (u^0 )^2 }{R} -1\right] } \,, \end{aligned}$$

with the upper sign for outgoing and the lower one for ingoing geodesics. The timelike Killing vector $\xi ^a =\left( \partial /\partial t \right) ^a$ guarantees the conservation of the energy per unit mass of the test particle *E* along these geodesic curves:

29 $$\begin{aligned} E =- g_{ab} \, \xi ^a u^b = \frac{|C_0| u^0}{R} =\text{ const. } \,, \end{aligned}$$

where $$u^0 >0 $$ because these curves are future-oriented, hence *E* is strictly positive. Equation (28) then gives

30 $$\begin{aligned} u^1 = \pm \, \frac{2}{\sqrt{R}} \, \sqrt{ \left( R_\mathrm {max}-R\right) \left( \frac{E^2 R}{|C_0|} -1 \right) } \,, \end{aligned}$$

which tells us that:

- For a given energy *E* determined by the initial condition $\left( R_0, \dot{R}_0 \right)$, radial motion is only possible if
  31 $$\begin{aligned} R > R_\mathrm {min}\equiv \frac{|C_0|}{E^2} \end{aligned}$$
  (otherwise $u^1$ becomes imaginary). Ingoing radial motion stops at $R_\mathrm {min}$ and a test particle cannot approach the origin, which is consistent with the fact that, according to Eq. (29), $u^0=ER/|C_0| \rightarrow 0$ as $R\rightarrow 0$.
- Outgoing radial motion stops at $R_\mathrm {max}$, where $u^1$ vanishes for both outgoing and ingoing radial geodesics, and a particle starting initially at $R_\mathrm {max}$ remains there irrespective of its initial energy.
- Since *R* is limited by $R_\mathrm {max}$, the possible energies are bounded from below,
  32 $$\begin{aligned} E > \sqrt{ \frac{|C_0|}{ R_\mathrm {max} }} = \frac{2 |C_0|}{ \sqrt{C_0^2 C_3^2 +4 C_0 C_2}} \equiv E_\mathrm {min} \,; \end{aligned}$$
  particles with energy below, or equal to, the minimum threshold $E_\mathrm {min}$ do not move.

### Case $C_2 \ne 0$, $C_3=0$

In this case we are left with only two parameters $\left( C_0, C_2 \right)$. Now $g(r) = C_0 C_2 -r^2$, which requires

33 $$\begin{aligned} C_0 C_2>0 \,, \quad \quad 0\le r \le \sqrt{C_0 C_2} \,. \end{aligned}$$

The areal radius is

34 $$\begin{aligned} R(r) =\frac{g(r)}{|C_0|}= \frac{C_0 C_2-r^2}{|C_0|} \end{aligned}$$

with $r=0$ corresponding to $R=C_0C_2/|C_0|$, while $r=\sqrt{C_2 \, \text{ sign }(C_0) }$ corresponds to the origin $R=0$ of the physical radial coordinate. The areal radius *R*(*r*) varies in the range

35 $$\begin{aligned} 0\le R \le C_2 \,\text{ sign }(C_0) =|C_2| \end{aligned}$$

(where, in the last equality, we used the fact that $$C_0C_2>0$$) and is a decreasing function of *r* since $$dR/dr=-2r/|C_0| $$ is always negative in the allowed range. Inverting the relation between radial coordinates, one obtains

36 $$\begin{aligned} r(R)= \sqrt{ C_0 C_2 -|C_0|R} \end{aligned}$$

which, in conjunction with

37 $$\begin{aligned} dr = -\frac{|C_0| dR}{2\sqrt{ C_0 C_2 -|C_0|R}} \end{aligned}$$

yields the line element

38 $$\begin{aligned} ds^2 = -\frac{|C_0|}{R} \, dt^2 +\frac{ dR^2}{4\left( \frac{|C_2|}{R} - 1\right) } \, + R^2 d\varOmega _{(2)}^2 \,. \end{aligned}$$

This geometry is the same as that of the previous case $C_1=0$, $C_3 \ne 0$ given by the line element (16), but now $R_\mathrm {max}=|C_2|$. Again, the energy density is non-zero and the Ricci scalar diverges at the origin $R=0$.

### Case $C_2=0 \,, C_3 \ne 0$

For these parameter values, $g(r)= r\left( C_0 C_3 -r \right)$ requires $C_0 C_3$ to be positive and, therefore, we have the range $0\le r \le C_0 C_3$ of the Buchdahl radius. Correspondingly, the areal radius

39 $$\begin{aligned} R(r) = \frac{r\left( C_0 C_3-r\right) }{|C_0|} \end{aligned}$$

varies in the interval

40 $$\begin{aligned} 0 \le R \le \frac{ |C_0| C_3^2}{4} \,, \end{aligned}$$

beginning from zero at $r=0$, increasing to the maximum

41 $$\begin{aligned} R_\mathrm {max} \equiv R \left( \frac{C_0 C_3}{2} \right) = \frac{ |C_0| C_3^2}{4} \,, \end{aligned}$$

and then decreasing until it vanishes again at $r=C_0 C_3$. There are two coordinate charts $0\le r \le C_0 C_3/2$ and $C_0 C_3/2 \le r \le C_0 C_3$ covering the same physical region $0 \le R \le R_\mathrm {max}$ and we restrict ourselves to the former, in which $dR/dr>0$. Equation (39) yields

42 $$\begin{aligned} r^2 -C_0C_3 r +|C_0|R=0 \end{aligned}$$

with roots

43 $$\begin{aligned} r(R) =\frac{1}{2} \left( C_0 C_3 \pm \sqrt{ C_0^2 C_3^2 -4|C_0|R} \, \right) \,, \end{aligned}$$

where we choose the lower sign for consistency with $dR/dr>0$ and $0\le r\le C_0C_3/2$. Then $g(r)=|C_0|R$ and

44 $$\begin{aligned} dr=\frac{|C_0|}{\sqrt{ C_0^2 C_3^2 -4|C_0|R}} \, dR \end{aligned}$$

give the line element

45 $$\begin{aligned} ds^2 = -\frac{|C_0|}{R} \, dt^2 + \frac{dR^2}{4\left( \frac{ R_\mathrm {max} }{R} - 1 \right) } \, +R^2 d\varOmega _{(2)}^2 \end{aligned}$$

which is the same as the line element (16), but with $R_\mathrm {max}$ now given by Eq. (41). The Ricci scalar

46 $$\begin{aligned} {\mathcal {R}} =\frac{8\kappa }{5} \, \rho = \frac{8\kappa ^2}{R^2} = \frac{8\kappa ^2 C_0^2}{r^2\left( C_0 C_3-r\right) ^2 } \,, \end{aligned}$$

diverges at the origin $R=0$ (which corresponds to $r=0$ in the chart with $dR/dr>0$), therefore there is a naked spacetime singularity there.

## General solution $C_1\ne 0$

The line element for the generic family of Semiz solutions is (3) [1]. The presence of four parameters with relatively wide ranges now makes it difficult to reach definite conclusions and we focus on special cases.

### $C_3=0$ is Schwarzschild

When $C_3 =0$, the energy density (5) and the pressure $P=-\rho /5$ vanish identically and this spacetime is empty. Since the geometry is also spherically symmetric and asymptotically flat (as we are going to show) it must be the Schwarzschild one, according to the Jebsen-Birkhoff theorem [15]. In fact, we have $f(r) = 3\left( C_1 C_2+r \right)$, the areal radius is

47 $$\begin{aligned} R=\frac{C_1C_2 +r}{|C_1|} \,, \end{aligned}$$

and

48 $$\begin{aligned} C_0+C_1r= C_1 |C_1|R +C_0-C_1^2 C_2 \,, \end{aligned}$$

then $dr=|C_1|dR$, yielding the line element

49 $$\begin{aligned} ds^2&=-\frac{ C_1^2 R +\left( C_0 -C_1^2 C_2\right) \,\text{ sign }(C_1)}{R} \, dt^2 \nonumber \\&\quad + \frac{C_1^2R}{C_1^2 R +\left( C_0-C_1^2 C_2\right) \,\text{ sign }(C_1)} \, dR^2 +R^2 d\varOmega _{(2)}^2 \nonumber \\ \end{aligned}$$

50 $$\begin{aligned}&\simeq -d\bar{t}^2 +dR^2 +R^2 d\varOmega _{(2)}^2 \quad \quad \text{ as } \, R\rightarrow + \infty \,, \end{aligned}$$

where $d\bar{t} \equiv |C_1| dt$. This geometry is asymptotically flat: by introducing the constant

51 $$\begin{aligned} m \equiv \frac{1}{2C_1^2}\left( C_1^2 C_2-C_0\right) \, \text{ sign }(C_1) \end{aligned}$$

(which is not necessarily positive) and rescaling the time coordinate according to $t \rightarrow \bar{t} = |C_1| \, t$, the line element (49) is written as the Schwarzschild one

52 $$\begin{aligned} ds^2=-\left( 1-\frac{2m}{R} \right) d\bar{t}^2 +\frac{dR^2}{1-2m/R} +R^2 d\varOmega _{(2)}^2 \end{aligned}$$

describing a black hole if $m>0$ and a naked central singularity if $m<0$.

### Special case $C_2=0$

We have three parameters $\left( C_0, C_1, C_3 \right)$ with $C_1C_3 \le 0$ and now $f(r)= 3r+C_3\left( C_0 + C_1 r\right) ^3$; the areal radius is

53 $$\begin{aligned} R(r)= \frac{ 3r +C_3\left( C_0+C_1 r \right) ^3}{3|C_1|} \,. \end{aligned}$$

We have

54 $$\begin{aligned} \frac{dR}{dr}= \frac{1}{|C_1|} \left[ 1- |C_1C_3| \left( C_0 +C_1r \right) ^2 \right] \,, \end{aligned}$$

which is positive for

55 $$\begin{aligned} \left| r+\frac{C_0}{C_1} \right| < \frac{1}{|C_1| \sqrt{ |C_1 C_3|}} \,. \end{aligned}$$

To proceed, let us consider the situation $r\ge -C_0/C_1$, in which case *R* increases in the interval

56 $$\begin{aligned} r_\mathrm {min} \equiv -\frac{C_0}{C_1} \le r \le \frac{1}{|C_1| \sqrt{ |C_1C_3|}} -\frac{C_0}{C_1} \equiv r_\mathrm {max} \end{aligned}$$

with $$ R_\mathrm {min} \le R \le R_\mathrm {max} $$ and

57 $$\begin{aligned}&R_\mathrm {min} \equiv R\left( r_\mathrm {min} \right) = \frac{-C_0}{C_1|C_1|} \end{aligned}$$

58 $$\begin{aligned}&R_\mathrm {max} \equiv R \left( r_\mathrm {max} \right) = \frac{1}{3|C_1|} \left[ \frac{3}{|C_1| \sqrt{|C_1 C_3|}}-\frac{ 3C_0}{C_1} \right. \nonumber \\&\quad \left. + C_3\left( \frac{ \text{ sign }(C_1)}{ \sqrt{|C_1 C_3|} } \right) ^3 \right] = \frac{1}{3|C_1|} \left[ \frac{3 + \, \text{ sign }(C_1 C_3) }{|C_1| \sqrt{|C_1 C_3|} }-3\frac{ C_0}{C_1} \right] \nonumber \\&\qquad = \left\{ \begin{array}{lll} \frac{1}{3|C_1|} \left( \frac{2}{|C_1| \sqrt{|C_1 C_3|}} -\frac{3C_0}{C_1} \right) &{} \quad \text{ if } &{} C_1 C_3<0 \,,\\ &{}&{}\\ \frac{1}{C_1^2} \left[ \frac{1}{\sqrt{|C_1 C_3|}} -C_0 \, \text{ sign }(C_1) \right] &{} \quad \text{ if } &{} C_1 C_3 =0 \,. \end{array} \right. \end{aligned}$$

We have again a compact space. Rewriting the line element (3) in terms of the areal radius produces a cumbersome expression that does not depend only on *R* but contains also *r*(*R*) because the relation *R*(*r*) cannot be inverted explicitly.

### The even more special case $C_0=C_2=0$

In this case we have only two parameters $\left( C_1, C_3 \right)$, $f(r)= r\left( 3+C_1^3 C_3 r^2 \right)$, and the areal radius is

59 $$\begin{aligned} R(r)= & {} \frac{f(r)}{3|C_1|}=\frac{ 3r+ C_1^3 C_3 r^3}{3|C_1|} \nonumber \\= & {} \frac{r \left[ 3+ (C_1 C_3)C_1^2r^2 \right] }{3|C_1| } \le \frac{r}{|C_1|} \,, \end{aligned}$$

where the last inequality follows from $C_1 C_3 \le 0$. Since

60 $$\begin{aligned} \frac{dR}{dr} =\frac{ 1- |C_1 C_3| C_1^2 r^2}{|C_1|} \ge 0 \quad \forall r\in \left( 0, \frac{1}{ |C_1| \sqrt{ |C_1 C_3|} } \right) \,, \end{aligned}$$

the areal radius is an increasing function of *r* in the interval $\left( 0, \frac{1}{|C_1| \sqrt{ |C_1 C_3|}} \right)$ with $R(0)=0$, is maximum at $\frac{1}{ |C_1| \sqrt{ |C_1 C_3|}}$ and then decreases, vanishing again at $r= \sqrt{ \frac{3}{|C_1 C_3| C_1^2}}$. This compact space corresponds to the range

61 $$\begin{aligned} 0 \le R \le R_\mathrm {max} = \frac{2}{3C_1^2 \sqrt{ |C_1C_3|} } \end{aligned}$$

of the areal radius, with $R \simeq r/|C_1|$ as $r\rightarrow 0^{+}$. Equation (59) is inverted by first obtaining

62 $$\begin{aligned} C_3 C_1^3 r^3+3r-3|C_1|R=0 \end{aligned}$$

and then solving for

63 $$\begin{aligned} r= \frac{ \left[ A(R)\right] ^{1/3} }{2 C_3 C_1^2}-\frac{2}{C_1 \left[ A(R)\right] ^{1/3} } \end{aligned}$$

where

64 $$\begin{aligned} A(R)=\left( 12 |C_1|C_1 R + 4\sqrt{ 9 C_1^4 R^2 +4 } \, \right) C_1^2 C_3^2 \,, \end{aligned}$$

while the two remaining roots are imaginary. Substituting the relation between differentials

65 $$\begin{aligned} dr=\frac{|C_1|}{1+C_3 C_1^3 r^2} \, dR \end{aligned}$$

and using

66 $$\begin{aligned} 1 + C_1^3 C_3 r^2=\frac{ \left[ A(R)\right] ^{2/3}}{4C_1 C_3}+\frac{4C_1 C_3}{\left[ A(R)\right] ^{2/3}}-1 \end{aligned}$$

yield the line element

67 $$\begin{aligned} ds^2&= -\frac{1}{|C_1| R}\left\{ \frac{ \left[ A(R)\right] ^{1/3} }{2C_3} - \frac{2C_1}{\left[ A(R)\right] ^{1/3} } \right\} dt^2 \nonumber \\&\quad + \frac{C_1^2|C_1|R}{B(R)} \, dR^2 + R^2 d\varOmega ^2_{(2)} \,, \end{aligned}$$

where

68 $$\begin{aligned} B(R)&= \frac{ \left[ A(R) \right] ^{5/3}}{32 C_1^2 C_3^3 }-\frac{32 C_1^3 C_3^2 }{ \left[ A(R)\right] ^{5/3} } + \frac{5 \left[ A(R)\right] ^{1/3} }{2C_3}-\frac{3A(R) }{8 C_1 C_3^2} \nonumber \\&\quad + \frac{24 C_1^2 C_3 }{A(R)} -\frac{10 C_1}{ \left[ A(R) \right] ^{2/3}} \,. \end{aligned}$$

Again, the many combinations of parameters and the cumbersome metric coefficients do not lend themselves to a straightforward and transparent analysis, but it is clear that also in this case we have a compact 3-space of finite extent.

Using (3.20), the energy density (5) reduces to

69 $$\begin{aligned} \rho (R)= & {} -5P(R)= \frac{5 {\mathcal {R}}}{8\kappa } = - \frac{5\kappa \, C_1^3 C_3 r^2}{R^2} \nonumber \\\approx & {} - \frac{5\kappa }{R^2} \left[ (C_1 C_3)^{1/3} +\frac{1}{(C_1 C_3)^{1/3} } -2 \right] \,,\nonumber \\&\end{aligned}$$

as $R\rightarrow 0^{+}$. Hence, $\rho$ and *P* are singular at the origin, together with the Ricci scalar ${\mathcal {R}}$ and

70 $$\begin{aligned} {\mathcal {R}}_{ab} {\mathcal {R}}^{ab} = \frac{28 \kappa ^2}{25} \, \rho ^2 \,. \end{aligned}$$

As $R\rightarrow 0^{+}$, we have the asymptotics

71 $$\begin{aligned} A(R)&\approx 8C_1^2 C_3^2 \,, \end{aligned}$$

72 $$\begin{aligned} B(R)&\approx \left( C_1^4 C_3 \right) ^{1/3} -\frac{7}{2 \left( C_1 C_3^4 \right) ^{1/3} } + 5 \left( \frac{C_1^2}{C_3} \right) ^{1/3} -3C_1\nonumber \\&\quad +\frac{3}{C_3} \equiv B_0 \,, \end{aligned}$$

73 $$\begin{aligned} g_{00}&\approx -\frac{1}{|C_1|R} \left[ \left( \frac{C_1^2}{C_3} \right) ^{1/3} - \left( \frac{C_1}{C_3^2} \right) ^{1/3} \right] \,, \end{aligned}$$

74 $$\begin{aligned} g_{11}&\approx \frac{ C_1^2 |C_1| R}{B_0} \,, \end{aligned}$$

and $g_{00} \rightarrow \infty$ while $g_{11} \rightarrow 0$ as $R\rightarrow 0$.

## Conclusions

We have studied the nature of the new classes of static and spherically symmetric solutions of the Einstein equations given recently in Ref. [1] when the matter source is a perfect fluid with equation of state $P=-\rho /5$. The analytical solutions of Ref. [1] that we analyzed (except for the Schwarzschild solution obtained for $C_3=0$) describe compact spaces with naked central singularities. The “general” family of solutions (3) and (5) always reduces to Schwarschild when the parameter $C_3$ vanishes. In most other situations, the presence of three or four parameters and/or the cubic nature of the function *R*(*r*) hamper a complete description of the geometry. However, in all cases analyzed, except for the empty spacetime associated with $C_3=0$, we find a compact space of finite volume (a feature mentioned in [1]).

The fact that the geometry, together with the energy density and the pressure, is singular at $R=0$ is not necessarily the death knell for these solutions. In fact, it is deemed acceptable for fluid solutions of the Einstein equations to only model limited regions of relativistic stars, a procedure that is reflected in the authoritative Ref. [13] and in the more specialized literature. Indeed, even Newtonian stars are rarely modelled with a single fluid, corresponding to the fact that different regions at different temperatures and densities are described by different equations of state unless the stellar material is well mixed, which only happens in certain types of stars. Therefore, there is in principle the (physically well motivated) possibility of excising the singularity and replacing it with a more realistic geometry sourced by matter with a different equation of state. However, if one wants to describe a stellar interior with this exotic fluid, one must match it with an asymptotically flat Schwarzschild exterior. The fact that the solutions of [1] describe compact spaces points to a possible analogy with the Oppenheimer-Snyder model of gravitational collapse to a black hole [33]. In this model, a compact, positively curved Friedmann-Lemaître-Robertson-Walker universe collapsing to a Big Crunch is matched to a Schwarzschild exterior on the surface of a 2-sphere of symmetry [33], satisfying the Darmois-Israel junction conditions [34, 35]. However, in the Oppenheimer-Snyder model the matching is possible because the collapsing interior universe is filled by a dust with zero pressure everywhere. It is well known that the matching to a Schwarzschild exterior can only be done on a surface on which the pressure *P*(*R*) vanishes, otherwise the junction conditions are violated and there is a material layer on the matching surface, which is certainly not an ingredient of realistic stellar models. (This fact is highlighted in many studies of relativistic fluid balls [36–43] and fireballs [44].) However, for the fluid solutions of [1] under discussion, the pressure *P*(*R*) never vanishes. Therefore, the best that one could do is modelling a limited region of a stellar interior with the Semiz solutions for $P=-\rho /5$. To be physical, this region should correspond to a positive Misner-Sharp-Hernandez mass $M_\mathrm {MSH}$ and, therefore, should be sufficiently far away from the singularity at $R=0$. The excised region containing the origin should be modelled with a different, non-singular, solution of the Einstein equations.^1^ Then, the $w=-1/5$ solution should be matched continuously with another “intermediate” solution with non-vanishing pressure on a surface of constant radius, and the pressure in this layer should then go to zero at larger radii to make it possible to match it to a Schwarzschild exterior, satisfying again the Darmois-Israel junction conditions. In the absence of a specific need for such an involved “star” model in astrophysics, we will not pursue this object further, limiting ourselves to pointing out the constraints for such a construction. Probably some of the phenomenology unveiled here for the geometries found in [1] also applies to other classes of perfect fluid solutions of the Einstein equations. Whether this is the case will be established in future work.

## Data Availability

This manuscript has no associated data or the data will not be deposited. [Authors’ comment: There are no data associated with this article because of its theoretical and formal nature.]

## Footnotes

1 There is a subtlety: matching a Semiz region with another fluid solution is in principle possible because the Semiz geometries solve the Einstein equations with zero cosmological constant $\Lambda$. A non-zero $\Lambda$ would be present in all spacetime regions and matching one of them with a Schwarzschild exterior is impossible [45].

## References

[1] İ. Semiz, arXiv:2007.08166 [gr-qc]

[2] DeBenedictis A, Garattini R, Lobo FSN (2008). Phys. Rev. D.

[3] Chapline G (2004). eConf.

[4] Lobo FSN (2006). Class. Quant. Grav..

[5] Bilic N, Tupper GB, Viollier RD (2006). JCAP.

[6] Chan R, da Silva MFA, Villas da Rocha JF (2009). Gen. Relativ. Gravit..

[7] Yazadjiev SS (2011). Phys. Rev. D.

[8] Rahaman F, Maulick R, Yadav AK, Ray S, Sharma R (2012). Gen. Relativ. Gravit..

[9] Horvat D, Marunović A (2013). Class. Quant. Grav..

[10] Bhar P (2021). Phys. Dark Univ..

[11] Armendariz-Picon C, Lim EA (2005). JCAP.

[12] Amendola L, Tsujikawa S (2010). Dark Energy: Theory and Observations.

[13] Stephani H, Kramer D, MacCallum M, Hoenselaers C, Herlt E (2003). Exact Solutions of the Einstein Field Equations.

[14] Delgaty MSR, Lake K (1998). Comput. Phys. Commun..

[15] Wald RM (1984). General Relativity.

[16] V. Faraoni, *Cosmological and Black Hole Apparent Horizons*, Lect. Notes Phys. **907** (Springer, New York, 2015)

[17] Misner CW, Sharp DH (1964). Phys. Rev..

[18] Hernandez WC, Misner CW (1966). Astrophys. J..

[19] Hawking SW (1968). J. Math. Phys..

[20] Hayward SA (1994). Phys. Rev. D.

[21] Hayward SA (1996). Phys. Rev. D.

[22] Faraoni V, Giusti A (2020). Symmetry.

[23] V. Faraoni, A. Giusti, T.F. Bean, Phys. Rev. D **103**, 044026 (2021)

[24] I.Z. Fisher, Zh. Eksp, Teor. Fiz. **18**, 636–640 (1948)

[25] Bergmann O, Leipnik R (1957). Phys. Rev..

[26] Janis AI, Newman ET, Winicour J (1968). Phys. Rev. Lett..

[27] Buchdahl HA (1972). Int. J. Theor. Phys..

[28] Wyman M (1981). Phys. Rev. D.

[29] Dionysiou DD (1982). Astrophys. Space Sci..

[30] Agnese AG, La Camera M (1985). Phys. Rev. D.

[31] Virbhadra KS (1997). Int. J. Mod. Phys. A.

[32] Faraoni V, Giusti A, Fahim BH (2021). Phys. Rept..

[33] Oppenheimer JR, Snyder JR (1939). Phys. Rev..

[34] Darmois G (1927). Les Equations de la Gravitation Einsteinienne.

[35] W. Israel, Nuovo Cimento B **44**, (1966)1 *Errata* **48**, (1967) 463(E)

[36] Vaidya PC (1968). Phys. Rev..

[37] Mashhoon B, Partovi MH (1980). Ann. Phys. (NY).

[38] Srivastava DC, Prasad SS (1983). Gen. Relativ. Gravit..

[39] Thompson AH, Whitrow WJ (1967). Mon. Not. R. Astron. Soc..

[40] Thompson AH, Whitrow WJ (1968). Mon. Not. R. Astron. Soc..

[41] Bondi H (1969). Mon. Not. R. Astron. Soc..

[42] Bondi H (1967). Nature.

[43] Faraoni V, Atieh F (2020). Phys. Rev. D.

[44] Smoller J, Temple B (1998). SIAM. J. Appl. Math..

[45] Faraoni V, Jose S, Leblanc A (2022). Phys. Rev. D.

